# *Bacillus subtilis* utilizes the DNA damage response to manage multicellular development

**DOI:** 10.1038/s41522-017-0016-3

**Published:** 2017-03-24

**Authors:** Kevin Gozzi, Carly Ching, Srinand Paruthiyil, Yinjuan Zhao, Veronica Godoy-Carter, Yunrong Chai

**Affiliations:** 1 Department of Biology, Northeastern University, Boston, MA 02115 USA; 2 College of Forestry resources and environment, Nanjing Forestry University, Nanjing, 210037 China

## Abstract

Bacteria switch between free-living and a multicellular state, known as biofilms, in response to cellular and environmental cues. It is important to understand how these cues influence biofilm development as biofilms are not only ubiquitous in nature but are also causative agents of infectious diseases. It is often believed that any stress triggers biofilm formation as a means of bacterial protection. In this study, we propose a new mechanism for how cellular and environmental DNA damage may influence biofilm formation. We demonstrate that *Bacillus subtilis* prevents biofilm formation and cell differentiation when stressed by oxidative DNA damage. We show that during *B. subtilis* biofilm development, a subpopulation of cells accumulates reactive oxygen species, which triggers the DNA damage response*.* Surprisingly, DNA damage response induction shuts off matrix genes whose products permit individual cells to stick together within a biofilm. We further revealed that DDR^ON^ cells and matrix producers are mutually exclusive and spatially separated within the biofilm, and that a developmental checkpoint protein, Sda, mediates the exclusiveness. We believe this represents an alternative survival strategy, ultimately allowing cells to escape the multicellular community when in danger.

## Biofilm disruption: A bacterial escape plan

Exposure to chemicals that damage DNA makes *Bacillus subtilis* bacteria turn off genes that maintain the biofilm state, releasing some bacteria. This mechanism to free cells from a biofilm is surprising, as biofilms holding bacteria together are generally considered to form as a protective mechanism for the bacteria. Yunrong Chai and colleagues at Northeastern University in Boston, USA, detected the surprising behavior in biofilms exposed to superoxide—a chemical species that damages DNA. They suggest that when the potential for damage to bacterial DNA reaches a threshold it signals that the environment is not suitable for cells to remain in the entrapped biofilm form, triggering disruption of the biofilm as a survival strategy. This response to DNA damage may be relevant to clinical conditions as biofilms are involved in many infectious diseases.

## Introduction

Biofilms are bacterial multicellular communities ubiquitously present in nature,^1, 2^ and are a primary cause of hospital-acquired infections.^3^ The Gram-positive soil-dwelling bacterium *Bacillus subtilis* is a model system for biofilm studies.^4^ Examples of biofilms in *B. subtilis* are floating pellicles at the air–liquid interface in liquid cultures, structurally complex colonies on solid surfaces, and plant root-associated biofilms in the rhizosphere.^4–6^ Biofilm formation is a multicellular developmental life cycle, in which genetically identical bacterial cells differentiate and adopt phenotypically distinct cell types, likely to increase the fitness of the entire community.^4, 7^ The biofilm is held together by an extracellular matrix, which facilitates spatial organization of the multicellular community. In *B. subtilis*, the matrix consists of exopolysaccharides,^8^ protein fibers (TapA and TasA),^9^ and a hydrophobin (BslA).^10^ Signals for induction of *B. subtilis* biofilm assembly derive from either the environment, such as plant-released polysaccharides and surfactin-like molecules,^5, 11, 12^ or from cellular metabolic activities, such as serine starvation or acetate.^13, 14^

Reactive oxygen species (ROS) are an important metabolic signal primarily generated during aerobic bacterial growth.^15^ In eukaryotic organisms, ROS accumulation is linked to aging.^16^ Additionally, ROS are found in the environment or released from hosts as a defense mechanism.^17^ ROS, such as hydrogen peroxide and superoxide, can cause damage to macromolecules such as DNA, protein, and lipids, and trigger multiple cellular stress responses.^17, 18^ In this study, we show that ROS exert a strong negative impact on biofilm development, and that this effect is mediated by the DNA damage response (DDR).

## Results

**Fig. 1:**
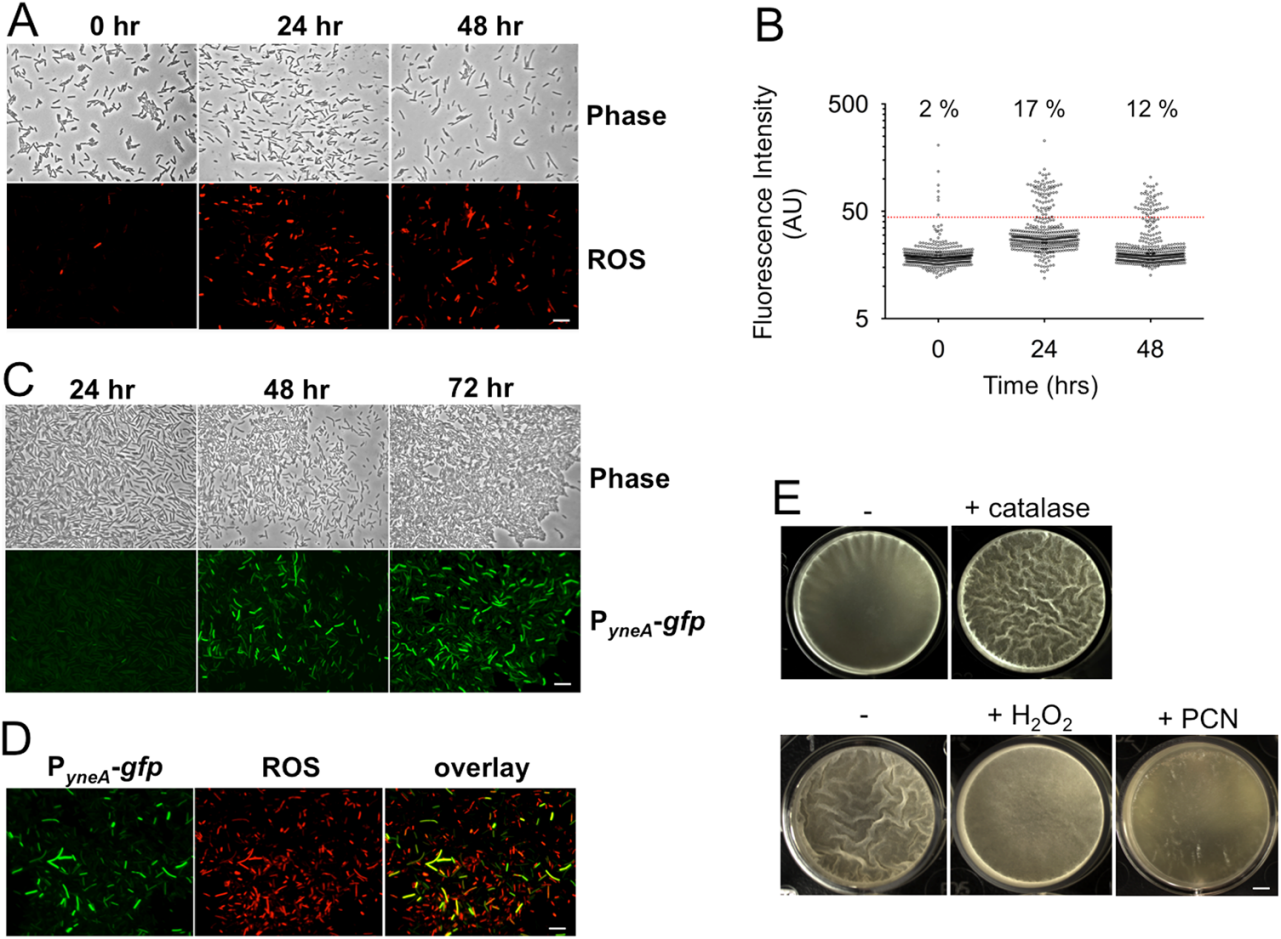
ROS accumulation triggers the DNA damage response (DDR) in a subpopulation of cells in a *B. subtilis* biofilm. **a** Older biofilms accumulate ROS. *B. subtilis* NCIB 3610 cells collected from 0, 24, and 48 h biofilm pellicles were treated with a superoxide-specific dye and observed under fluorescent microscopy. Cells with strong superoxide accumulation show a *red color*. Scale bar represents 10 μm. **b** There is an increase in ROS accumulation in cells from older biofilms. Quantification of cells from **a** was performed using ImageJ.^44^ Each *dot* represents one cell and the *y*-axis shows fluorescent intensity. The *dotted horizontal line* indicates the threshold used to define ROS^ON^ cells (described in Methods). **c** Older biofilms accumulate DNA damage. *B. subtilis* YCN036 cells containing the P_*yneA*_-*gfp* DDR reporter were collected from 24, 48, and 72 h pellicle biofilms and observed under fluorescent microscopy (*green cells*). Scale bar represents 10 μm. **d** ROS^ON^ cells and DDR^ON^ cells overlap. Same cells applied in **c** were collected from 48 h pellicle biofilms and stained with superoxide-specific dye (*red cells*). An overlay image of ROS^ON^ cells (in *red*) and DDR^ON^ cells (in *green*) is shown. Scale bar represents 10 μm. **e** ROS negatively impacts *B. subtilis* biofilm development. In the *top panels*, the *B. subtilis* 3610 biofilm pellicles treated with catalase (1 mg ml^−1^) was more robust than the one without treatment. Images were taken after 24 h of incubation. *Bottom panels* show that the pellicle biofilms treated with H_2_O_2_ (0.001%, v/v) or pyocyanin (PCN) (2.5 μg ml^−1^) are much weaker than without treatment. Images were taken after 48 h of incubation. Scale bar represents 2.5 mm

The importance of ROS in bacterial cell physiology has been well studied, but primarily in the context of free-living cells.^18, 19^ Here, we are interested in understanding the influence of both endogenous and exogenous ROS on *Bacillus subtilis* biofilm development. We note that diffusion and turnover of ROS may differ significantly between the biofilm and the free-living environment.^20^ By application of a superoxide (O_2_^−^)-specific fluorescent dye, we observed an increasing accumulation of ROS in a subpopulation of cells in early and mature biofilms (Fig. 1a–b, 24 and 48 h). The proportion of ROS^ON^ cells (stained in red; Fig. 1a–b) vs. total cells in a pellicle biofilm increased from ~2% at hour 0 (initial inoculum of exponential phase cells) to ~17% at hour 24 (early biofilm). After 48 h (mature biofilm), there was a mild decrease in the ratio of ROS^ON^ cells (12%, Fig. 1a–b).

Since ROS cause oxidative DNA damage,^19^ we wondered whether the accumulation of ROS was associated with an induction of the DDR. In *B. subtilis*, oxidative DNA damage leads to formation of single-stranded DNA, which is bound by RecA, the multi-function recombinase, to form RecA-nucleoprotein filaments, whose co-protease activity promotes auto-proteolysis of LexA, the DDR master repressor.^21^ Thus, DNA damage leads to induction of LexA repressed genes such as *yneA*, which encodes a protein antagonist to the cell-division protein FtsZ, causing elongated cells with arrested cell division.^22^ A fluorescent transcriptional DDR reporter, P_*yneA*_-*gfp*, was constructed and used to assess DDR induction in *B. subtilis*. Pellicle biofilms of this reporter strain were collected over time and observed under fluorescent microscopy. We observed strong DDR induction (green) in a subset of cells from both 48 and 72 h biofilm samples (Fig. 1c). Furthermore, when we simultaneously probed ROS accumulation within the biofilm, we found that DDR^ON^ cells (in green) were largely inclusive in the ROS^ON^ cells (in red) (Fig. 1d). In general, >90% of the cells with strong DDR induction (Fig. 1d, bright green cells) stained ROS positive in multiple independent experiments. In conclusion, our results suggest that strong ROS accumulation is associated with induction of the DDR in a subpopulation of cells in the *B. subtilis* biofilm.

To determine whether ROS accumulation influences biofilm development in *B. subtilis*, we tested the effect of hydrogen peroxide (H_2_O_2_) and the ROS-generating chemical pyocyanin (PCN) on biofilm robustness. Addition of exogenous H_2_O_2_ (0.001%, v/v), 10 times below the minimal inhibitory concentration (MIC, 0.01%, v/v) under our conditions, significantly decreased the robustness of mature pellicle biofilms (Fig. 1e). Another ROS-generating chemical, PCN produced by *Pseudomonas* sp.,^23^ had a similar effect at a concentration (2.5 μg ml^−1^) fourfold below the MIC (10 μg ml^−1^) that we determined (Fig. 1e). In the case that these sub-MIC concentrations of chemicals were affecting biofilm robustness via some minor effect on growth, we tested whether removing ROS would stimulate biofilm formation. In bacteria, superoxide (O_2_^−^) is converted to peroxide (H_2_O_2_) by superoxide dismutases, which is then converted to H_2_O by catalases.^19^ Addition of catalase (1 mg ml^−1^) stimulated earlier and more robust pellicle biofilm formation compared to untreated biofilms in the absence of catalase (Fig. 1e). Taken together, our results suggest that the level of ROS is inversely correlated with biofilm robustness in *B. subtilis*.

**Fig. 2:**
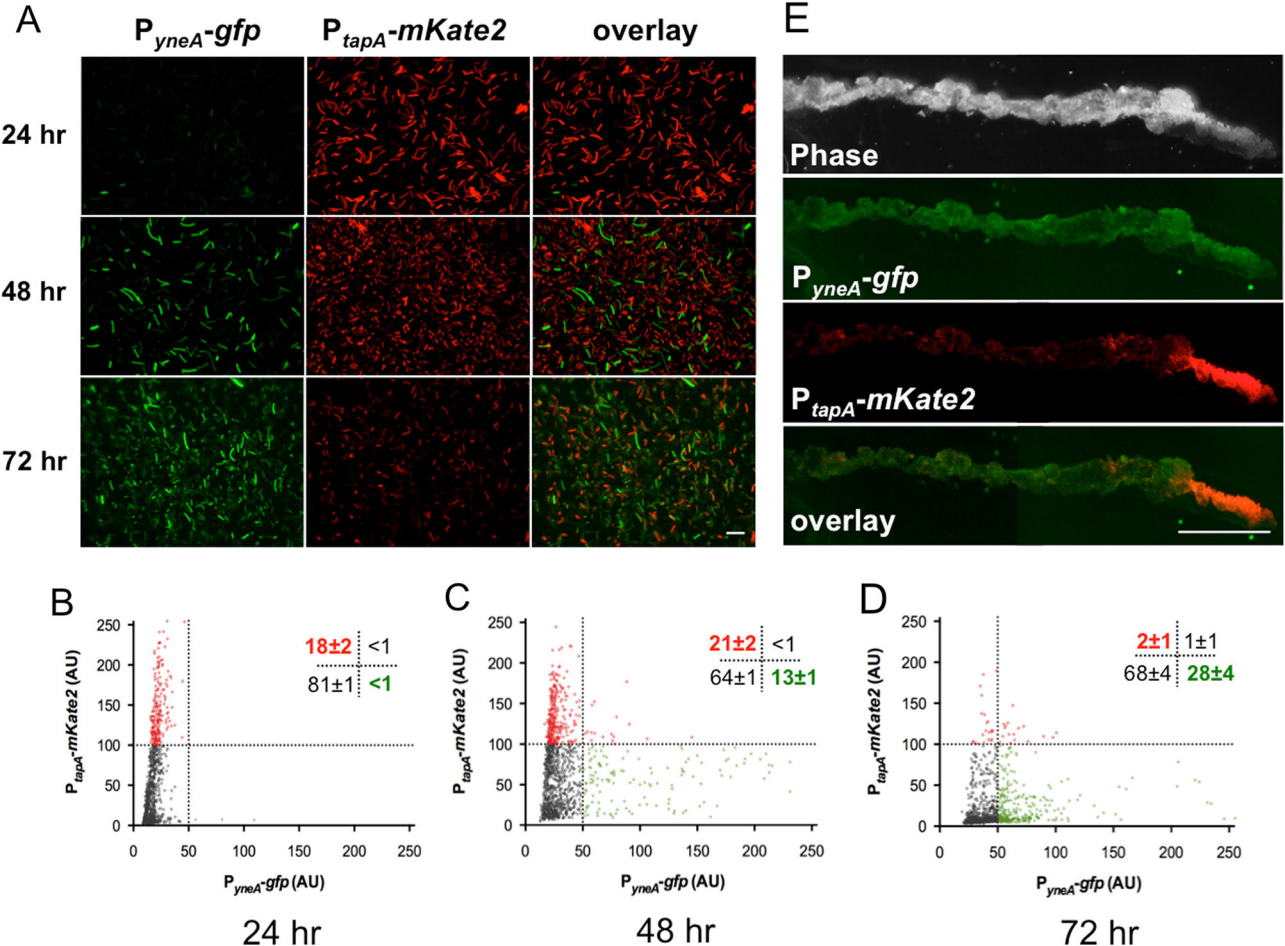
DDR^ON^ cells and matrix producers are mutually exclusive and spatially separated in the *B. subtilis* biofilm. **a** DDR^ON^ cells and matrix producers are mutually exclusive cell types. Pellicle biofilms by the dual reporter strain (YCN040: P_*yneA*_-*gfp*, *green*, DDR reporter; P_*tapA*_-*mKate2*, *red*, matrix reporter) were collected after 24, 48, and 72 h of incubation and examined under fluorescent microscopy for DDR (P_*yneA*_-*gfp*) and matrix gene expression (P_*tapA*_-*mKate2*). An overlay image of cells expressing the two reporters is shown. Scale bar represents 10 μm. **b**–**d** Quantification of DDR^ON^ and matrix producer cells. Statistic analyses of cells collected at 24, 48, and 72 h from **a** using MicrobeJ plugin for ImageJ.^44^ Each *dot* represents a cell, *y*-axis represents red fluorescent intensity (matrix) while *x*-axis represents green fluorescent intensity (DDR). The *horizontal* and *vertical dotted lines* represent the thresholds used to define DDR^ON^ and matrix producing cells. Provided data set represents one independent replicate. The values in the upright corner of each panel are the average percentage ratio with standard devation of cells that fell into the corresponding area in the plot, determined from three biological replicates. **e** DDR^ON^ cells and matrix producers have distinct spatial localization in the biofilm. Thin-section fluorescent microscopic analysis of a 48-h colony biofilm formed by the dual reporter strain showing spatial localization of DDR^ON^ cells (P_*yneA*_-*gfp*) and matrix producers (P_*tapA*_-*mKate2*). Scale bar represents 1 mm

Previous studies show that phenotypic heterogeneity occurs in *B. subtilis* biofilms and is critical for establishing mutually exclusive cell types.^4, 24, 25^ Since increasing ROS levels is associated with an inhibition of biofilm formation and that cells experiencing ROS accumulation overlapped with DDR^ON^ cells, we wondered how DDR^ON^ cells and matrix producers interplay with each other temporally and spatially in the biofilm. A strain bearing both a DDR reporter, P_*yneA*_-*gfp*, and a matrix reporter, P_*tapA*_-*mKate2*, was constructed to allow for observation of both DDR induction and matrix production at the single cell level. Intriguingly, we found that cells expressing the DDR reporter, and those expressing the matrix reporter, occupied separate subpopulations in the biofilm (Fig. 2a). Cells strongly expressing the DDR reporter (bright green) do not express the matrix reporter, as shown in overlay images (Fig. 2a, overlay panel), indicating that they were mutually exclusive. Furthermore, the proportion of DDR^ON^ cells and matrix-producers in the biofilm showed opposing temporal dynamics; the proportion of DDR^ON^ cells rose from 13 ± 1% at 48 h to 28 ± 1% at 72 h while the proportion of matrix producers declined over the same period of time from 21 ± 2 to 2 ± 1% (Fig. 2b–d).As controls, there is little non-specific background fluorescence detected in wild-type cells bearing no reporter (Fig. S1).

To further investigate spatial localization of DDR^ON^ cells and matrix producers, we applied thin-section fluorescent microscopy^26^ to a 48 h colony biofilm formed by the dual reporter strain. We found that DDR^ON^ cells (in green) localize throughout the biofilm colony, while matrix producers (in red) cluster at the edge of the colony (Fig. 2e). At the colony scale, we did observe some overlap of DDR^ON^ cells and matrix producers at the edge of the colony. It is possible that DDR^ON^ cells and matrix producing cells share this space while being mutually exclusive at the individual cell level as we observed previously in Fig. 2a. Unfortunately, we did not have the resolution to see individual cells in the colony thin-section experiment. Interestingly, in a previous study,^26^ it has been shown that matrix producers were seen to localize to a mid-layer, which spanned throughout the colony at 48 h. Here, we observed these cells at the edge of the colony after 48 h. We attribute the discrepancy to the different biofilm-inducing media used in the two studies (minimal media supplemented with 0.5% glycerol and 0.5% glutamate in the previous study vs. LBGM (LB supplemented with 1% glycerol and 100 μM MnSO_4_) in this study) and different biofilm maturation rates. Biofilm formation peaks by 48 h in LBGM, and an earlier time point corresponding to initial biofilm formation may have revealed a more widespread distribution of these cells. Overall, our results suggest that DDR^ON^ cells and matrix producers are mutually exclusive cell types that are spatially separated in the biofilm.

**Fig. 3:**
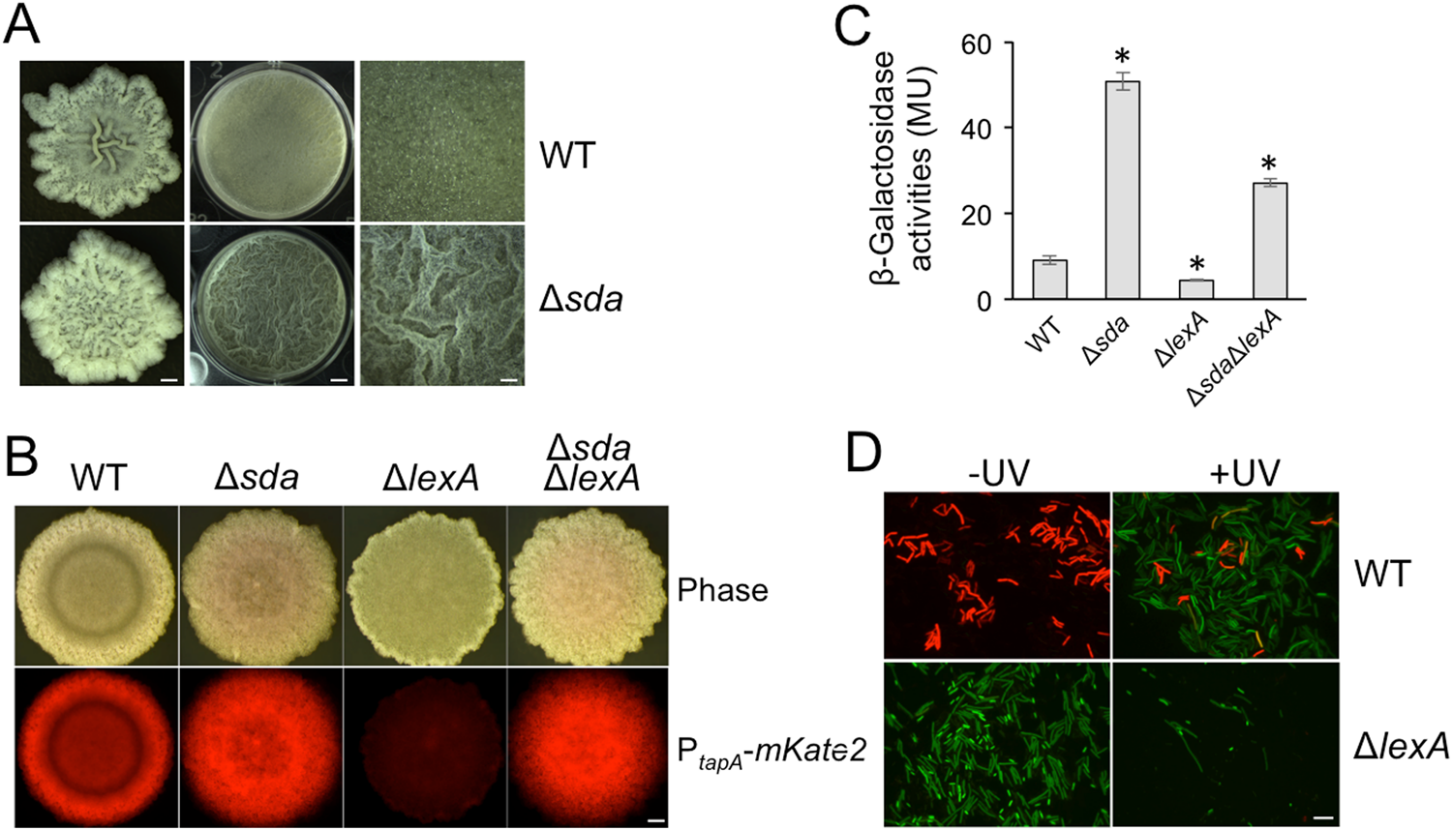
ROS negatively impacts biofilm development through the DDR induction. **a** The biofilm formed by a Δ*sda* strain is more robust than the isogenic parental strain. Comparison of the biofilm (both colony and pellicle) phenotypes between the parental strain (NCIB 3610) and the isogenic Δ*sda* mutant strain (YCN025). Right-hand panels show zoom-in images of the pellicle biofilms shown in the middle panels. Biofilms were incubated at 30 °C for 48 h before the images were taken. From *left* to *right*, scale bar represents 1, 2.5, and 0.6 mm, respectively. **b** Matrix gene expression is stronger and more uniform in colony biofilms of the Δ*sda* mutant strains. Colony biofilms formed by the wild-type strain, the Δ*sda*, the Δ*lexA*, and the Δ*sda* Δ*lexA* double mutant strains with the P_*tapA*_-*mKate2* reporter. Colony biofilms were grown on LBGM agar plates at 30 °C for 24 h prior to imaging. Scale bar represents 1 mm. **c** Quantification of matrix gene expression. *β*-Galactosidase activities of the P_*tapA*_-*lacZ* matrix reporter in the wild type, the Δ*sda* mutant, the Δ*lexA* mutant, and the Δ*sda* Δ*lexA* double mutant strains were assayed in LBGM shaking culture. Assays were performed in triplicate using mid-exponential cells and following published protocols.^25^ A two-tailed student’s *t*-test was used to determine statistical significance between each mutant and the wild-type strain (* indicates *p*-value < 0.05). **d** UV treatment triggered DNA damage lowered matrix gene expression. The dual reporter cells (P_*yneA*_-*gfp*, P_*tapA*_-*mKate2*) of the wild-type and the Δ*lexA* mutant strains were treated with UV light, and examined by fluorescence microscopy after 1 h. *Green cells* express the DDR P_*yneA*_-*gfp* reporter while *red cells* express the matrix P_*tapA*_-*mKate2* reporter. Scale bar represents 10 μm

We next wanted to investigate genetically how DDR^ON^ cells and matrix producers become mutually exclusive. The DDR regulon has been characterized in *B. subtilis*.^27^ One gene in the regulon, *sda*, encodes a developmental checkpoint protein.^27, 28^ Sda inhibits the phospho-transfer from the histidine kinase KinA to the phospho-relay protein Spo0F,^29^ a step required for activation of the sporulation master regulator Spo0A.^30^ This mechanism is likely to block sporulation prior to the repair of damaged DNA. We demonstrate here that Sda not only has a role in controlling sporulation, as has been previously shown,^28, 29^ but also in biofilm development as the Δ*sda* mutant showed a hyper-robust biofilm phenotype after 48 h of incubation (Fig. 3a). This was expected since Spo0A governs regulatory pathways for both sporulation and biofilm formation in *B. subtilis*.^4^ Meanwhile, the strain lacking the DDR master repressor (Δ*lexA*) has a weaker biofilm phenotype that we hypothesize is due to overexpression of *sda* (Fig. 3b). The Δ*sda* Δ*lexA* double mutant resembles a Δ*sda* strain, which provides further support that *sda* is downstream of *lexA* and mediates the effect of *lexA* on biofilm formation (Fig. 3b). In addition, the biofilm phenotype of both the Δ*lexA* and Δ*sda* mutations can be complemented with the respective wild-type genes in an ectopic locus (Fig. S2). Note that the differences in colony robustness for both WT and the Δ*sda* strain between Fig. 3b and Fig. 3a (and Fig. S2 as well) are due to different incubation times (24 h in Fig. 3b vs. 48 h in Figs. 3a and S2). When we measured matrix gene expression levels in these strains, we found that the matrix reporter(P_*tapA*_-*lacZ*) was expressed fourfold higher in the Δ*sda* strain (*p*-value < 0.001), but about twofold lower in the Δ*lexA* strain (*p*-value < 0.05) compared to wild type (Fig. 3c). Interestingly, the repression of matrix gene expression in the Δ*lexA* strain was only partially rescued in the double Δ*sda* Δ*lexA* mutant (*p*-value < 0.001); expression in the double mutant was still significantly lower than in the Δ*sda* strain (Fig. 3c *p*-value < 0.001), indicating that the DDR pathway may regulate matrix genes by an additional unknown mechanism. The same regulation was observed in the Δ*lexA*, Δ*sda*, and Δ*sda* Δ*lexA* strains bearing another matrix gene reporter, P_*tapA*_-*mKate2* (Fig. 3b). To summarize, we showed that ROS accumulate during biofilm formation, that ROS accumulation correlates with DDR induction, and that DDR induction in turn downregulates matrix gene expression via Sda.

In addition to ROS, we would expect a similar inverse regulation between the DDR genes and matrix genes in cells treated by other DNA damaging agents, such as ultraviolet (UV) light. Indeed, upon treatment with UV light, the DDR was strongly induced, shown by robust expression of the DDR reporter P_*yneA*_-*gfp* while the matrix reporter P_*tapA*_-*mKate2* was almost completely off (Fig. 3d). There were a few cells expressing both reporters (in yellow, Fig. 3d), which may be cells transitioning from matrix-producers to DDR^ON^ cells upon DNA damage. In a Δ*lexA* background, the P_*tapA*_-*mKate2* matrix reporter was off while the P_*yneA*_-*gfp* DDR reporter was constitutively expressed in the absence of DNA damage (Fig. 3d). These results reinforce the idea that DNA damage triggers inverse regulation on genes for the DDR and matrix production.

## Discussion

Bacteria constantly encounter and respond to various stresses from the environment or hosts. It is thus important for us to understand how bacteria cope with these stresses to survive. Here, we investigated the effect of oxidative stress on multicellular bacterial communities. Oxidative damage can be endogenously accrued from cell metabolism or toxins and result in damaged DNA.^17^ Specific ROS-triggered responsive pathways lead to upregulation of gene products, such as catalases, which eliminate ROS and alleviate oxidative damage.^18^ Additionally, accumulation of damaged DNA upregulates genes needed to repair DNA lesions through the more generalized DDR.^31^ However, despite a great deal of understanding in free living bacteria,^18, 31^ the response of bacterial communities as a whole to DNA damage is not as well understood.

In this study, we showed that superoxide (a proxy for total ROS) accumulates in a subset of cells in a *B. subtilis* biofilm, and this accumulation is associated with an induction of the DDR (Fig. 1). Remarkably, induction of the DDR in those cells was shown to lead to a significant negative regulation of biofilm formation genes (Figs. 2–3). We hypothesize that for *B. subtilis*, there is a critical threshold of DNA damage, which signals that the surrounding environment is not suitable to remain sessile in a biofilm. Decreased matrix production in response to DNA damage would likely permit increased biofilm dispersal, allowing cells in the community to escape the stressful environment. This strategy might be evolutionarily advantageous for *B. subtilis*, which usually establishes symbiotic mutualism with its plant host.^5^ Thus, this mechanism may allow *B. subtilis* to evaluate the safety of the environment, such as a plant root host.

This finding in *B. subtilis* may seem counter intuitive since biofilm formation has been shown to be a defense mechanism to protect bacteria from environmental stresses.^32–34^ However, upon severe DNA damage, bacteria may have to make a “fight or flight” decision; either enforce the biofilm for protection or degrade the biofilm in order to escape. This decision may vary depending on the species and situation. For some bacteria, when exposed to DNA damage, it might be advantageous to stick together and form stronger biofilms for protection, while for others, it might be better to escape to find a more favorable niche. *B. subtilis* represents an example of a bacterium whose DDR induction leads to repression of biofilm formation genes. When ROS are produced endogenously from cell metabolism as a by-product of aging, remaining in the biofilm may be suicidal as the biofilm environment may prevent diffusion and effective turnover of ROS. A future investigation would be to track temporal and spatial ROS accumulation within a biofilm, which may further shed light on differential metabolic states of cells within a biofilm. Additionally, elucidating the mechanism by which only a subset of cells accumulate ROS was beyond the scope of the present work, but it remains an interesting future direction.

Cell-fate heterogeneity is perceived as a beneficial strategy for *B. subtilis* as cells specialize and fill niches within the biofilm to enhance the fitness of the entire community. During biofilm development, cells will eventually break down the matrix to allow for dispersal from aged biofilms. However, the signal triggering biofilm dispersal has yet to be understood. In this work, we present that ROS accumulation and DNA damage are associated with decreased matrix gene expression in a subset of cells. DNA damage may serve as an initial trigger to transition from biofilm assembly to biofilm disassembly. This mechanism also may let cells to switch out of a cell fate allowing for transition to another cell fate.

In summary, we showed that the DDR is induced as a biofilm ages, and DDR genes influence biofilm formation. We hypothesize that when oxidative stress is too strong and causes severe DNA damage, it indicates that the surrounding environment is not suitable for *B. subtilis* to remain sessile. This may serve as a mechanism for *B. subtilis* to sense when resources have been exhausted (signaled by endogenous ROS accumulation) as well as if the host plant is not suitable for root colonization. Broadly, we believe these findings may demonstrate a strategy that allows a subset of bacterial cells to turn off matrix production and subsequently escape from communal living upon sensing severe environmental stress.

## Methods

### Strains and media

A list of strains, plasmids, and oligonucleotides used in this study are included in Table S1. *B. subtilis* strain NCIB 3610^6^ and derived strains were cultured in lysogenic broth (LB) at 37 °C. Biofilm formation was induced in *B. subtilis* using LBGM.^35^ Biofilms were incubated at 30 °C for the indicated times. Enzymes were purchased from New England Biolabs. Chemicals and reagents were purchased from Sigma or Fisher Scientific. Oligonucleotides were purchased from Integrated DNA Technologies (IA, USA) and DNA sequencing was performed at Genewiz (NJ, USA). Antibiotics, if needed, were applied at the following concentrations: 10 μg ml^−1^ of tetracycline, 1 μg ml^−1^ of erythromycin, 100 μg ml^−1^ of spectinomycin, 20 μg ml^−1^ of kanamycin, and 5 μg ml^−1^ of chloramphenicol for transductions and transformation in *B. subtilis* and 100 μg ml^−1^ of ampicillin for *E. coli* DH5α transformations.

### Strain construction

The insertional *sda*::*erm* and *lexA*::*erm* deletion mutants (BKE25690 and BKE17850, respectively) were purchased from the *Bacillus* Genetic Stock Center (BGSC, http://www.bgsc.org) and introduced into NCIB 3610 via SPP1 phage-mediated transduction^8, 36^ to generate YCN025 and YCN020, respectively. To allow for combination of *sda* deletion mutation with the *lexA*::*erm* knockout, an insertional *sda*::*tet* deletion mutant was constructed using long-flanking homology PCR, which has been described previously,^37^ and using primers sda-P1-4 (Table S2).The PCR product was introduced by transformation into *B. subtilis* PY79 and transformants were selected for double-crossover recombination on LB agar plates supplemented with appropriate antibiotics. The deletion mutation was confirmed by PCR amplification of the locus and DNA sequencing. The deletion mutation of *sda* (*sda*::*tet*) was introduced into the *lexA*::erm mutant and other genetic backgrounds by SPP1-mediated transduction as described previously, resulting in various double mutants and derivative reporter strains listed in Table S1.

Published protocols were followed for general methods of molecular cloning.^38^ To construct the promoter-*gfp* fusions, the promoter of *yneA* was amplified by PCR using genomic DNA from the wild-type strain NCIB 3610^6^ as template and using primers P_*yneA*_-F1 and P_*yneA*_-R1 (Table S2). The purified PCR product was cloned into the *Eco*RI and *Hin*dIII restriction sites of a plasmid pYC121, which bears a promoter-less *gfp* gene (*gfp-mut*2) flanked by the *amyE* sequences.^25^ The resulting plasmid was cloned into and then purified from *E. coli* DH5α and introduced by transformation into the *B. subtilis* laboratory strain PY79 using a standard *B. subtilis* transformation protocol.^39^ Transformants were selected for double-crossover recombination at the chromosomal *amyE* locus on LB agar plates with appropriate antibiotics and by verification of loss of amylase activities on LB + starch plates as previously shown.^40^ This reporter fusion was then transferred into the NCIB 3610 strain to generate the reporter strain YCN036 using SPP1 phage-mediated transduction, as described previously.^36, 40^ The P_*tapA*_-*mKate2* reporter fusion integrated at the *sacA* chromosomal locus was transferred from the strain TMN503 (ref. 41) to NCIB 3610 and YCN036 using SPP1 phage-mediated transduction to generate strains YCN095 and YCN040, respectively. To generate strains YCN050, and YCN098 to YCN101, SPP1 phage-mediated transduction was used to transfer Δ*lexA*::erm from YCN020 to YCN040,FC591, YCN095, YCN098, and YCN099, respectively. To generate the complementation strains of the Δ*lexA*::erm and Δ*sda*::erm mutants, the region containing the promoter (200 and 244 bp upstream of the start codon, respectively) through the stop codon of the coding sequence was PCR amplified using the NCIB 3610 genomic DNA as the template and using primers lexA-compF, lexA-compR, and sda-compF, sda-compR, respectively. These PCR fragments were digested with *Bam*HI and *Eco*RI and were cloned into the same sites of the *amyE* integration vector pDG1662.^42^ The plasmids were purified from *E. coli* DH5α and were then introduced by transformation into *B. subtilis* PY79 as described above. SPP1 phage-mediated transduction was used to transfer these constructs into the *amyE* locus of YCN020 and YCN025, respectively, generating strains YCN120 and YCN121, respectively. To construct reporter strains with the P_*tapA*_-*lacZ* transcriptional fusion, the parent strain FC591,^43^ which is a 3610 derivative bearing the P_*tapA*_-*lacZ* at the *amyE* locus, was used. The transcription fusion from FC591 was introduced into various 3610 derivative strains by SPP1 phage-mediated transduction.

### Colony thin-section

Thin-section of the colony biofilms was done using modified published protocols.^26^ The colony biofilm was developed on the LBGM plate for 48 h and was excised from the agar plate, placed in a Tissue-Tek Cryomold vinyl specimen mold (#4565, VWR), embedded in Tissue-Tek O.C.T. Compound (#4583, VWR) and frozen with liquid nitrogen for 10 min. The sample was then longitudinally cut into 10-μm-thick slices using a Microm HM 560 cryosectioner set at −20 °C with an Edge Rite knife. The thin sections were placed onto VWR Superfrost Plus Micro Slides (#48311-703) and air-dried. A drop of PBS was applied to each sample followed by application of a cover slip. Samples were immediately imaged under fluorescent microscopy (see details below).

### Microscopic imaging and ROS staining

For colony thin-sectioning imaging, samples were imaged at 2× magnification using a Leica MZ10 F dissecting microscope (Model: MSV269) and a Leica DMC3000G camera. The gray-scale fluorescent images were artificially colored using ImageJ (Version 1.46r).^44^ Thin sections of wild-type colonies bearing no fluorescent reporter were imaged to determine background fluorescence. Sections of strains only bearing either the P_*yneA*_-*gfp* DDR reporter or the P_*tapA*_-*mKate2* matrix reporter were imaged to identify level of bleed-through between the two channels. For colony biofilms, samples were imaged at 4× magnification using the Leica MSV269 dissecting microscope with a Leica DFC2900 camera. The same exposure and acquisition settings were used for each colony.

For single-cell fluorescence imaging, cells were cultured as described above. After incubation for 24, 48, or 72 h, the pellicles were collected and disrupted with mild pipetting. One milliliter samples were mildly sonicated with 5-second pulses at the 1.5 output scale for three times (Branson, Model W185), pelleted at 14,000 rpm for 1 min, and washed once with PBS. We performed live/dead staining to confirm that there was no noticeable cell lysis caused by mild sonication by using a commencially available kit (L7012, ThermoFisher). For cell imaging, 1 μl of the PBS resuspension was placed on a 1% agarose pad and covered with a cover slip. Cells from three independent biological replicates were imaged using a Leica DFC3000 G camera on a Leica AF6000 microscope. Non-specific background fluorescence was determined by quantifying WT cells bearing no reporter. Imaging of samples collected from different time points was conducted using the same exposure settings. Single-cell fluorescence was quantified on >600 cells per replicate using the MicrobeJ plugin for ImageJ.^44^ The expression for both reporters was analyzed and binned into bright (P_*yneA*_-*gfp* > 50 units and P_*tapA*_-*mKate2* > 100 units) and dim (P_*yneA*_-*gfp* < 50 units and P_*tapA*_-*mKate2* < 100 units) populations. For green fluorescent proteins (GFP) observation, the setting of the excitation wavelength was 450–490 nm, while the setting of the emission wavelength was 500–550 nm. For mKate2 observation, the excitation wavelength setting was at 540–580 nm and the emission wavelength setting was at 610–680 nm.

Superoxide accumulation was measured using the Total ROS/Superoxide Detection Kit from Enzo Life Sciences (ENZ-51010). Pellicle samples were harvested as described above. One-milliliter samples were incubated with 0.4 μl of the provided superoxide dye for 30 min, prior to fluorescent imaging. Cell fluorescence was quantified for approximately 400 cells for each time point using MicrobeJ.^44^ For *B. subtilis*, cells with a fluorescence of ≥2 standard deviations above the mean of *T*_0_ cells were determined to be ROS^ON^.

### Biofilm assays in *B. subtilis*

For colony biofilm formation, cells were grown to exponential phase in LB broth and 2-μl of the culture was spotted onto LBGM plates solidified with 1.5% agar. The plates were incubated at 30 °C for 2–3 days. For pellicle biofilm formation, cells were grown to exponential phase in LB broth, and 4-μl of the culture was inoculated into 4-ml of LBGM liquid medium in a 12-well microtiter plate (VWR). Plates were incubated 24–48 ≥ h at 30 °C. For treatment of catalase, peroxide or PCN, the compound was diluted to the concentrations indicated in the figure legends and added to the liquid medium. Colony and pellicle images were taken as described above using a Leica MSV269 dissecting scope and a Leica DMC2900 camera.

### UV light treatment

Late exponential phase cultures of YCN040and YCN050 were diluted 1:100 in LBGM and grown for 3 h at 37 °C (until mid-exponential phase). For UV treatment, 3-ml of the cultures were pelleted at 14,000 rpm and resuspended in an equal volume of PBS. Two-ml samples were evenly placed in an empty sterile glass petri dish. Samples were irradiated in the dark 0.75 m from a UV germicidal lamp, resulting in 54 J m^−2^ as measured by a model UVX digital radiometer (UVP, Inc.). Parallel samples were prepared the same way but left untreated. After UV treatment cells were centrifuged, resuspended in 3 ml of fresh LBGM, and outgrown for 1 h at 37 °C with or without shaking followed by fluorescent imaging.

### MIC determination

To determine the MIC for H_2_O_2_ and PCN, mid-exponential phase cultures of NCIB 3610 were diluted 1:100 in LBGM containing a range of concentrations of either H_2_O_2_ or pycoyanin. Cultures were then grown overnight with shaking at 37 °C. The minimal concentration yielding no growth was determined to be the MIC for the compound. MICs were confirmed in conditions used for pellicle biofilm growth.

## Electronic supplementary material

Figure S1
