# Supplementary material for: Bacillus subtilis utilizes the DNA damage response to manage multicellular development
Source: NPJ Biofilms Microbiomes. 2017 Mar 24;3:8. doi: 10.1038/s41522-017-0016-3 (PMC5445613; doi:10.1038/s41522-017-0016-3)
Supplement: Supplementary file 1 — Figure S1 [file 41522_2017_16_MOESM1_ESM.pdf]

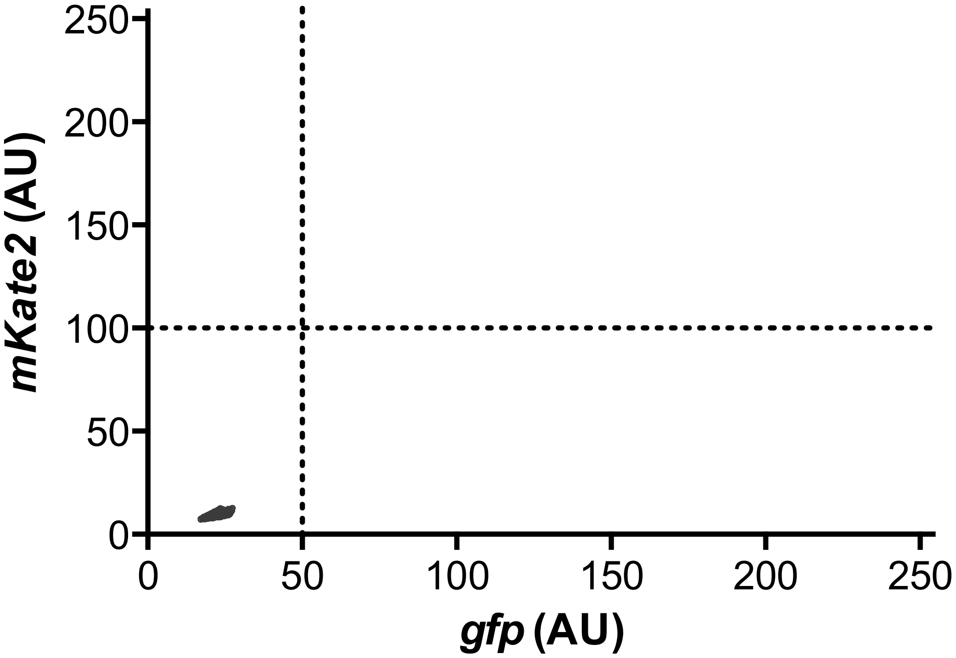

Figure S1

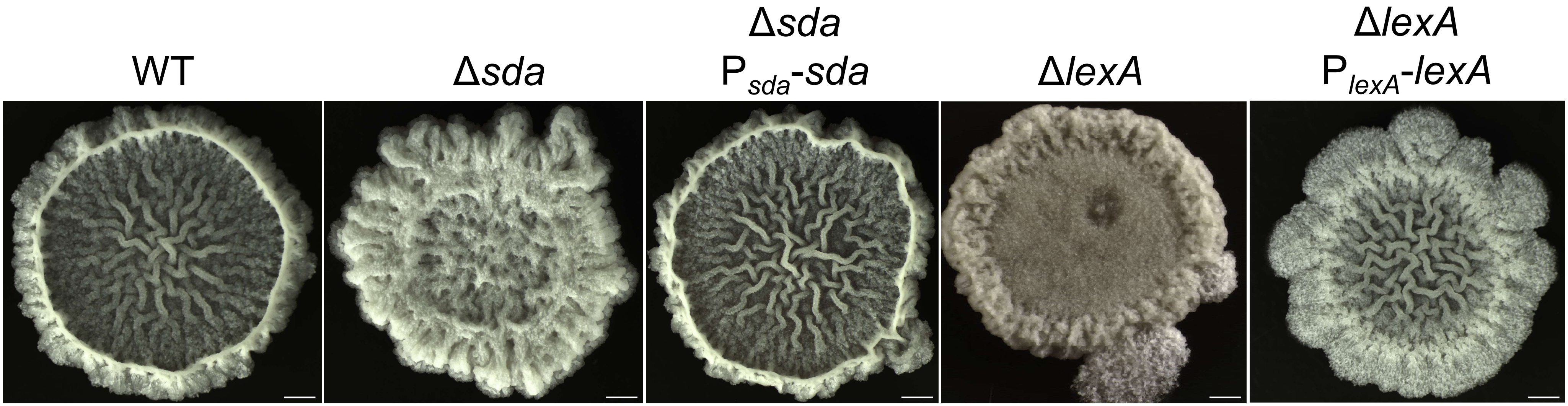

Figure S2

Supplemental Figure Legends

Fig S1. Background fluorescence of *B. subtilis* in pellicle biofilms. Pellicle biofilms of NCIB 3610 cells were collected after 24 hours of incubation and were imaged with fluorescence microscopy as described in the Methods. Fluorescence of cells on the GFP and mKate2 channels was quantified using MicrobeJ.

Fig. S2. Complementation of the *sda* and *lexA* deletion mutants show a rescue of biofilm architecture. Shown are colony biofilms formed by the wild type strain (NCIB 3610), the *sda* deletion mutant (YCN025), the complementation of the *sda* deletion mutant (YCN258), the *lexA* deletion mutant (YCN020), and the complementation of the *lexA* deletion mutant (YCN278) on LBGM agar. Images were taken after 2 days of incubation of the biofilms at 30°C. The scale bar represents 0.25 cm.

Supplemental Table 1: Strains and plasmids used in this study.

Strain	Parent	Genotype	Source
DH5α		An *E. coli* strain for molecular cloning	Invitrogen
PY79		A laboratory strain of *B. subtilis* for genetic transformation	(1)
168		A domesticated strain of *B. subtilis*	(2)
NCIB 3610		An undomesticated strain of B. subtilis capable of biofilm formation	(3)
BKE17850	168	Δ*lexA*::*erm*	BGSC
BKE25690	168	Δ*sda*::*erm*	BGSC
TMN503	3610	*sacA*::P_{tapA}-*mKate2*, kan^R	(4)
FC591	3610	*amyE*::P_{tapA}-*lacZ*, $spec^R$	(5)
YCN020	3610	Δ*lexA*::*erm*	This work
YCN025	3610	Δ*sda*::*erm*	This work
YCN036	3610	*amyE*::P_{yneA}-*gfp*, chl^R	This work
YCN040	YCN036	*sacA*::P_{tapA}-*mKate2*, kan^R, *amyE*::P_{yneA}-*gfp*, chl^R	This work
YCN050	YCN040	Δ*lexA*::*erm*, *sacA*::P_{tapA}-*mKate2*, kan^R, *amyE*::P_{yneA}-*gfp*:chl^R	This work
YCN095	3610	*sacA*::P_{tapA}-*mKate2*, kan^R	This work
YCN096	YC121	Δ*sda*::*tet*, *amyE*::P_{tapA}-*lacZ*, $spec^R$	This work
YCN097	YCN095	Δ*sda*::*tet*, *sacA*::P_{tapA}-*mKate2*, kan^R	This work
YCN098	FC591	Δ*lexA*::*erm*, *amyE*::P_{tapA}-*lacZ*, $spec^R$	This work
YCN099	YCN095	Δ*lexA*::*erm*, *sacA*::P_{tapA}-*mKate2*, kan^R	This work
YCN100	YCN098	Δ*lexA*::*erm* Δ*sda*::*tet*, *amyE*::P_{tapA}-*lacZ*, $spec^R$	This work
YCN101	YCN099	Δ*lexA*::*erm* Δ*sda*::*tet*, *sacA*::P_{tapA}-*mKate2*, kan^R	This work
YCN258	YCN020	Δ*sda*::*erm*, *amyE*::P_{lexA}-*lexA*, chl^R	This work
YCN278	YCN025	Δ*lexA*::*erm*, *amyE*::P_{sda}-*sda*, chl^R	This work
**plasmid**			
pYC121		An *amyE* integration vector with a promoter-less gfp(mut2), chl^R, amp^R	(6)
pDG1662		An *amyE* integration vector for complementation, chl^R, amp^R	(7)

Abbreviations for the antibiotic resistance genes: amp, ampicillin; chl, chloramphenicol; erm, erythromycin; kan, kanamycin; spec, spectinomycin; tet, tetracycline.

Supplemental Table 2: Oligonucleotides used in this study.

**Primers (5'>3')**	
lexA-compF	gtacggatccttaatggacggttctgaacacgc
lexA-compR	gtacgaattcaatcactgtaaacagaccgacaa
sda-compF	gtacgaattcgggtttcttcaccaggtgtgg
sda-compR	gtacggatccctattaagaagatacggaaataatatgtccgagcgatcttc
PyneA-F	gtcgaattcagcttcgtcatttttcgcacct
PyneA-R	gtcaagcttcaggaatgtttgttcgcattt
sda-P1	gtaactccgcacccgatacaaa
sda-P2	caattcgccctatagtgagtcgtgggagaggcacctccttaaaag
sda-P3	ccagcttttgttccctttagtgagtaataggaatttgtctattttt
sda-P4	gcagtgggtcaatatattaaaa

Supplemental references

1. **Schroeder JW, Simmons LA.** 2013. Complete Genome Sequence of Bacillus subtilis Strain PY79. Genome Announcements **1:**e01085-01013.

2. **Harwood CR, Wipat A.** 1996. Sequencing and functional analysis of the genome of Bacillus subtilis strain 168. FEBS Letters **389:**84-87.

3. **Branda SS, Gonzalez-Pastor JE, Ben-Yehuda S, Losick R, Kolter R.** 2001. Fruiting body formation by *Bacillus subtilis*. Proc Natl Acad Sci USA **98:**11621-11626.

4. **Norman TM, Lord ND, Paulsson J, Losick R.** 2013. Memory and modularity in cell-fate decision making. Nature **503:**481-486.

5. **Chu F, Kearns D, B. , McLoon A, Chai Y, Kolter R, Losick R.** 2008. A novel regulatory protein governing biofilm formation in *Bacillus subtilis*. Mol Microbiol **68:**1117-1127.

6. **Chai Y, Chu F, Kolter R, Losick R.** 2008. Bistability and biofilm formation in *Bacillus subtilis*. Mol Microbiol **67:**254-263.

7. **Guérout-Fleury AM, Frandsen N, Stragier P.** 1996. Plasmids for ectopic integration in *Bacillus subtilis*. Gene **180:**57-61.
